# Effects of Polyethylene Glycol on the Hydrogen Bonds and Stacking Interactions of DNA/DNA, DNA/RNA, and RNA/RNA Calculated by a Mesoscopic Model

**DOI:** 10.1021/acsomega.6c00104

**Published:** 2026-05-12

**Authors:** Luciano Gabriel Silva, Gerald Weber

**Affiliations:** Departamento de Física, 28114 Universidade Federal de Minas Gerais, Belo Horizonte, MG 31270-901, Brazil

## Abstract

The addition of polyethylene glycol (PEG) to the buffer of oligonucleotide melting experiments is a way to simulate the cellular crowding. PEG reduces the water activity, but how this affects the hydrogen bonding and base stacking is mostly unknown. Here, we use published melting temperature data for DNA/DNA (DD), DNA/RNA (DR), and RNA/RNA (RR) to calculate how the hydrogen bonds and stacking interactions change upon the addition of PEG. For this, we used a mesoscopic model that describes the helical stability in terms of the intramolecular interactions. For the hydrogen bonds, we find that PEG has almost no effect on DD and a moderate effect on CG in DR. However, for RR, hydrogen bonds become much stronger for CG but much weaker for AU. Changes in stacking interactions were also found to be strongest for RR.

## Graphical abstract

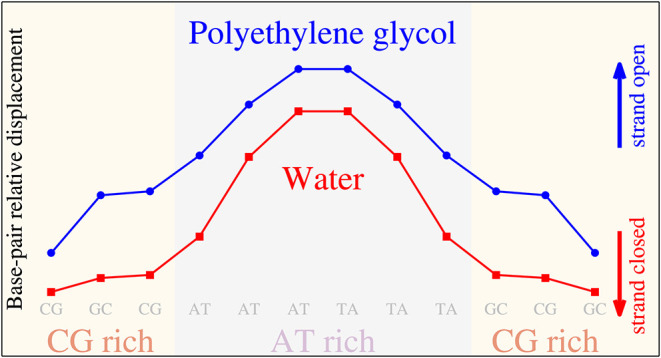

## 1 Introduction

Most experiments on oligonucleotide stability are performed in highly diluted aqueous solutions,^1^ which is entirely different from the intracellular environment.^2^ Approximately 20–40% of the cell volume is composed by biomolecules.^3–5^ In addition to biomolecules, cations are abundant within cells, playing important roles in regulating the biological functions of nucleic acids.^6–8^ The presence of macro and micromolecules in the cellular environment, usually called crowding, influences nucleotide stability. Crowding affects DNA in multiple ways, with the two main factors being water activity and exclusion volume.^9,10^ Water activity, the effective molar fraction of water available in solution,^11^ is changed through the interaction of organic micromolecules with water near nucleotides. Water molecules and solvated cations are the main factors responsible for reducing the energy difference between hydrogen bond formation and electrostatic repulsion of the phosphate group in the double helix.^12^ On the other hand, excluded volume increases the stability and melting temperature increases.^13–16^

A common method to mimic the effects of crowding in oligonucleotide melting experiments is the addition of a cosolute. Polyethylene glycol (PEG) is often chosen for this for being mostly inert and available in different molecular weights.^6,17,18^ Specifically, PEG with average molecular weight 200 (PEG200) simulates the effect of micromolecules on nucleotides. Due to its minute size, PEG200 presents a small exclusion volume impact, and its main effect is to reduce water activity. The polar part of PEG200 interacts through hydrogen bonds with water molecules. The slight displacement of water molecules caused by PEG is responsible for changing the hydration level around the double strands. Consequently, the stability of the strand is reduced and the denaturation temperature drops.^19–24^ It was also shown that the presence of PEG400 reduces the equilibrium constant of the DNA hybridization by 3 orders of magnitude and that PEG forms ion complexes that effectively reduces the amount of ions in the solution.^25^

Since PEG reduces the amount of water molecules in the vicinity of the oligonucleotide, let us discuss how water and cations help stabilize the nucleotide structure.^5^ When forming a DNA or RNA duplex with different combinations of nucleotides, such as AT and CG in DNA, the helix may become more distorted due to unequal forces between the base pairs. However, water molecules may reduce the helix distortion by destabilizing the hydrogen bonds between nucleotides.^10,26^ In addition, water molecules interact with the backbone, but more so with the phosphate groups,^10^ as well with cations present in the solution. Therefore, PEG interferes with the interactions between water molecules and nucleotides, pushing the system toward a single-stranded state and lowering the melting temperature.^6,7,19,27,28^ On the other hand, it was shown that PEG also results in ion complexation, effectively reducing the ionic strength of the buffer.^25^ In contrast, other types of solvents that also reduce water activity, such as ethanol, cause the cations to move toward the backbone and cause B-helix to A-helix transition in DNA.^29^ Therefore, reducing water activity alone does not fully explain the effect of PEG, and it is largely unknown how it affects the hydrogen bonds and stacking.

Few theoretical studies exists considering PEG200 interaction with oligonucleotides, only recently a molecular dynamics study of one specific DNA sequence interacting with PEG200 by Mathur and Singh^30^ has become available. However, we are presently not aware of similar studies for hybrid DNA/RNA and or RNA/RNA. Mesoscopic models, like the Peyrard-Bishop (PB) model, were used to study the effects of crowding in DNA as a confinement effect^31–34^ where an increase in melting temperature is observed; however, none of these cover the aspect of reduced water activity which destabilizes the oligonucleotide.

Mesoscopic models, like the PB model, use a set of effective potentials to describe the main interactions of the DNA double helix: the hydrogen bonds and the stacking interactions.^35^ These types of models, in combination with experimental melting temperatures, can be used to provide information about the intra- and intermolecular interactions present in nucleotides.^36^ The benefit of obtaining the parameters in this way is that they can be directly related to hydrogen bonds and stacking interactions. For instance, we were able to confirm NMR measurements^37,38^ showing that RNA AU has stronger hydrogen bond than DNA AT,^39^ while quantum-mechanical simulations could not.^40,41^ Additionally, the calculated parameters can be used to identify what may be happening in the system by comparing two or more distinct scenarios. For example, the PB model was capable of identifying a third hydrogen bond in a modified AT in LNA/DNA duplexes.^42^ Another important result was to describe the position of metals, Ag^+^ and Hg^2+^, between the mismatches CC and TT, respectively, when forming metal-mediated bonding.^43^ In both results, an increase in hydrogen bonding was observed compared with the unmodified situation. Another result of the model found was to find that varying the [Na^+^] concentration in saline solutions did not result in any significant change in the hydrogen bond of DNA duplexes.^44^ While the method has been clearly successful in describing a large range of experimental situations, it was not applied to systems with reduced water activity, such as oligonucleotides in PEG200.

Here, we apply our method of obtaining hydrogen bonds and stacking interactions from melting temperatures to oligonucleotides under the influence of PEG200. We use a set of published melting temperatures and perform a comparison between DNA/DNA (DD), RNA/RNA (RR), and hybrid DNA/RNA (DR). We show that PEG200 has little influence on the intramolecular interactions in DD, an intermediate effect on DR, while RR is strongly affected. In particular, we show that in PEG200 has a complex interaction with RR by strongly lowering the AU hydrogen bond while at the same time increasing the CG bond to a strength comparable to an LNA modification. To our knowledge, there is no previous work comprehensively comparing the hydrogen bonds and stacking interactions of the three major types of nucleic acids in the presence of PEG200.

## 2 Materials and Methods

### 2.1 Notation

As already mentioned in the introduction, we will refer to DNA/DNA duplexes as DD, RNA/RNA as RR, and hybrid DNA/RNA as DR. Throughout this work, we will use dA, dC, dG, and dT for DNA bases and rU, rG, rC, and rA for RNA. While it is common for hybrid DNA/RNA base pairs to be represented such as in dArT, this notation is less usual for nonhybrids in which case they will be represented such as dAdT (instead of AT for DD) and rArU (instead of AU for RR). This notation is adopted here for being less ambiguous, for instance, to readily distinguish RR CG (rCrG) from DD CG (dCdG).

Hydrogen bonds are represented by the pair participating in the bond, for instance, the dCdG pair for DD, dCrG pair for DR, and rCrG pair for RR. While the stacking interactions are represented by a (base pair 1)-(base pair 2) scheme. For example, the dCdG and dAdT pair, corresponding to the dimer 5′-d­(CA)-3′/3′-d­(GT)-5′, is represented by dCdG-dAdT. Note that due to symmetry considerations, 5′-d­(CA)-3′/3′-d­(GT)-5′ is equivalent to 5′-d­(TG)-3′/3′-d­(AC)-5′, therefore dCdG–dAdT is equivalent to dTdA–dGdC. For this work, we choose a notation based on alphabetical order, so we adopt dCdG–dAdT instead of dTdA–dGdC. The DR hybrid presents a special case where two distinct DNA and RNA strands come together to form a duplex. Therefore, hybrids are never symmetric. To represent a hybrid dimer, we could use two possible notations: 5′-d­(CA)-3′/3′-r­(GT)-5′ or 5′-r­(TG)-3′/3′-d­(AC)-5′, which are dCrG–dArT or rTdA–rGdC. For simplicity, in this work, we choose the notation starting with the deoxy base. However, occasionally, we may refer to RD as a hybrid where the RNA strand is the main strand such as in 5′-r­(TG)-3′/3′-d­(AC)-5′ for situations where such a distinction becomes necessary.

### 2.2 Melting Temperature Data Sets

A total of 36 melting temperatures for DD in PEG200 were taken from refs 20,21 shown in table S1. For DR hybrids in PEG200, we used 45 sequences and their melting temperatures from ref 45 (table S2), and for RR in PEG200 we used 45 sequences from ref 22 (table S3). All melting temperatures are for salt concentration Na^+^ of 122 mM, 40 wt % PEG200. The total strand concentration *C*_*t*_ was 200 μM for self-complementary and 100 μM for nonself-complementary sequences as established in ref 46. For the remaining details of buffer conditions, please see refs 20–22,45 .

A small set of melting temperatures, essentially replacing Na^+^ with K^+^, from Banerjee et al.^45^ for DR and Ghosh et al.^22^ for RR were used to verify the suitability of the new parameters in a similar way as in refs 22,45 . They are shown in Tables S4 and S5. Those temperatures were not used for parametrization.

### 2.3 Mesoscopic Model

The mesoscopic Peyrard-Bishop (PB) model describes the intramolecular interactions of oligonucleotides through simple potentials^35^ and by introducing a single variable *y* representing the displacement between the bases relative to their equilibrium distance. For example, stacking between the nearest-neighbors base pairs is described by a harmonic potential, while the hydrogen bond is represented by a Morse potential. For the *i*th base pair the Morse potential is

1 $$V\left(y_{i}\right)=D_{\alpha }{\left(e^{-y_{i}/\lambda_{\alpha }}-1\right)}^{2}$$

where the parameter *D*_α_ tells us how strongly the α base pair is hydrogen bonded, λ_α_ is related to the potential width, and *y*_*i*_ the relative displacement between the bases. For the nearest-neighbor stacking interaction, we use a modified harmonic potential which includes a small twist angle θ^36^

2 $$W\left(y_{i},y_{i-1}\right)=\frac{k_{\beta }}{2}\left(y_{i}^{2}-2y_{i}y_{i-1}\;\cos\theta +y_{i-1}^{2}\right)$$

where the parameter *k*_β_ is the coupling constant of nearest-neighbors of type β, and the angle is fixed at θ = 0.01 rad, which is introduced to overcome a well-known numerical problems with the transfer-integral technique.^47^

With the two potentials of eqs 1 and 2, we can calculate the classical partition function from which we obtain an adimensional index τ_*s*_(*P*), which is proportional to the melting temperature of each sequence *s*.^36^ Additionally, we can calculate the average strand displacement ⟨*y*_*i*_ ⟩ for each base pair *i* by calculating the expected value of *y* from the partition function.^36^

### 2.4 Calculation of Melting Temperatures

The index τ_*s*_(*P*) is used to predict melting temperatures for any sequence *s*, where *P* is a given set of parameters. Experimental melting temperatures *T*_*s*_ and the index τ_*s*_(*P*) are linearly dependent within groups of sequences of same length *N*

3 $$T\prime_{s}\left(P\right)=a_{0}\left(N\right)+a_{1}\left(N\right)\tau_{s}\left(P\right)$$

where *a*_0,1_(*N*) are the linear regression coefficients. These coefficients have a linear dependence with *N* ^1/2^

4 $$a_{k}\left(N\right)=b_{0,k}+b_{1,k}N^{1/2},\;k=0,1$$

and *b*_0,*k*_ and *b*_1,*k*_ are also linear regression coefficients.^36^

### 2.5 Parameter Optimization Procedure

The parameter optimization is carried out by minimizing the merit function

5 $$\chi_{j}^{2}\left(P_{j}\right)=\sum_{s=1}^{N}{\left[T_{s}-T\prime_{s}\left(P_{j}\right)\right]}^{2}$$

where *P*_*j*_ is the *j*-th tentative set of parameters. The goal is to find the best set of *P* parameters such that the calculated temperatures, *T*′_*s*_(*P*_*j*_), are closest to the experimental temperatures, *T*_*s*_. Considering that the set *P* contains *M* parameters, the numerical problem consists of finding the *M*-dimensional minima of eq 5, which is numerically implemented with a downhill simplex algorithm.^48^

In total, *M* = 12 parameters were varied for DD and RR sets: two Morse potential factors *D* and 10 stacking factors *k*; and *M* = 20 parameters were varied for DR sets, four *D* and 16 *k*. The number of parameters used for the DR hybrid is higher, since the symmetry reduction is not applicable.

Another equation considered in our discussion to evaluate the quality of the prediction is the average melting temperature deviation

6 $$\left\langle \Delta T\right\rangle =\frac{1}{N}\sum_{i=1}^{N}\left|T_{s}-T\prime_{s}\left(P_{j}\right)\right|$$

One of the main challenges in the numerical minimization of eq 5 is the unavoidable occurrence of local minima, that is, local valleys that do not coincide with the best overall minima. To overcome this problem, we repeat the minimization process multiple times, restarting from different initial parameters, until all average parameters converge with a difference of less than 0.1%. For each minimization round, the initial set of parameters *P*_init._ are randomly sampled around the 20% of value of seed parameters *P*_seed_, that is, within the interval of 0.8 *P*_seed_ to 1.2 *P*_seed_. Only Morse potentials *D* and stacking potentials *k* were varied. The λ parameters were kept constant since we have found, in previous studies, that their effect on melting temperatures is negligible.^36^

### 2.6 DR Low Salt (DR-LS) Parameters

In our previous work on DR, only high salt parameters were calculated.^49^ Therefore, we do not have low salt DR parameters for the mesoscopic model, unlike DD and RR where such parameters are already well established.^50,51^ The low salt DR parameters are needed to properly evaluate the changes due to the presence of PEG200. Here, we follow a similar procedure as in ref 49, but using the melting temperatures from refs 52–56 shown in table S6. We used the same generic seed parameters from de Oliveira Martins et al.,^49^ *D*_dArU_ = *D*_dTrA_ = 30 meV, *D*_dCrG_ = *D*_dGrC_ = 80 meV and a uniform *k* = 2.5 eV/nm^2^ for all 16 stacking factors. In total, two rounds of optimizations were calculated, with 500 minimizations per round, each time with different initial parameters. In the first round, we determined the *D* and *k* parameters for hybrids. The second and final round was used to estimate the influence of experimental uncertainty on our new parameters. A temperature uncertainty of 0.4 °C was used, which was obtained from the average reported uncertainty for each of the published measurements.^52–56^

### 2.7 No Cosolute (NC) Reference Set of Melting Temperatures

We generated a set of melting temperatures with no cosolute (NC) for the same DD, DR, and RR sequences for which we have temperatures in PEG200. For this we use the NN-model enthalpy and entropy parameters since they allow us to calculate these temperatures for the same total strand concentrations. We use the following NN parameters: SantaLucia et al.^57^ for DD, Banerjee et al.^56^ for DR, and Ferreira et al.^58^ for RR. Supporting Information tables S1–S3 show the NC melting temperatures, *T*′(NC), that were calculated in this way.

### 2.8 PEG200 Optimized Parameters

The optimization to obtain Morse and stacking potential parameters in the presence of PEG200 was calculated separately for the DD, DR, and RR sequences. For each type of oligonucleotide we proceeded as follows. First, we joined the experimental melting temperatures in PEG200 with the NC melting temperatures generated from their respective NN parameters (described in the previous section). We then calculated the optimized parameters by varying only the PEG200 Morse and stacking potentials but keeping the NC parameters constant at the same values of the NC reference. Both PEG200 and NC predicted temperatures were calculated from the same regression eqs 3 and 4. In this way we guarantee that the new PEG200 parameters are comparable to the reference NC. For the next three rounds, we remove the NC melting temperatures and now optimize only over the PEG200 temperatures. The last minimization round was to estimate the influence of experimental uncertainty on our new parameters. This was done by changing the experimental temperatures by small random amounts such that the resulting standard deviation corresponds to the experimental uncertainty. A temperature uncertainty of 0.7 °C was used, which was obtained from the average reported uncertainty for each of the published measurements.^20–22,45^ The final average melting temperature difference was ⟨Δ*T* ⟩, eq 6, was 0.77 °C for DD, 1.02 °C for DR, and 0.70° for RR.

### 2.9 Average Strand Opening

Using the new parameters, it is now possible to calculate the average strand opening for a given model temperature. This is the displacement of the base pairs from their equilibrium positions. Here, we calculate the average strand opening ⟨*y*_*i*_⟩ for each *i*th base pair in a sequence using the procedure detailed in references 42,59 .

### 2.10 Availability

All parameters and data sets presented here were included in the latest version of our free TfReg software^48^ which can be used to verify our results. The software and the parameters are available at http://tinyurl.com/tfregufmg, for other download options, see the Supporting Information.

## 3 Results and Discussion

We will discuss the results in the presence of PEG by comparing them to those in the absence of cosolutes and at similar salt concentration. For DD and RR, the Morse and stacking potentials are already available, since they were calculated in earlier works from our group.^50,51^ However, for DR, only high salt parameters were calculated in ref 49. Therefore, to be able to compare our results in PEG, it was necessary to perform this calculation for low salt (LS) concentrations. This new parametrization, which we call DR-LS, was carried out in a similar way as reported in ref 49, but using the 73 melting temperatures at low salt concentration collected from refs 52–56 . This is basically the data set ref 60 expanded with the sequences from Banerjee et al.^56^ and removing four sequences from Wang and Kool^61^ since they were measured in a buffer containing Mg^2+^. In Table 1, we show the new Morse potentials for DR in low salt. We observe a widening difference between dArU and dTrA, 19 meV compared to 12 meV for HS. For dCrG and dGrC, we notice a reversal with dGrC showing a higher Morse potential than dCrG, in contrast to the HS results. This change breaks the trend of lower stability of deoxypurine that we had observed for HS.^49^ For the stacking interaction parameters, shown in Table 2, they are very similar to HS except for dArU-dCrG which is much stronger. In Figure 1, we show the average displacement profiles for some sequences from ref 62 with varying deoxypyrimidine (dPy) content. In general, the opening profiles are similar for the three dPy contents, even for 100% dPy, Figure 1c, where we enlarged the vertical scale by 1 order of magnitude compared to Figure 1a.

**Figure 1:**
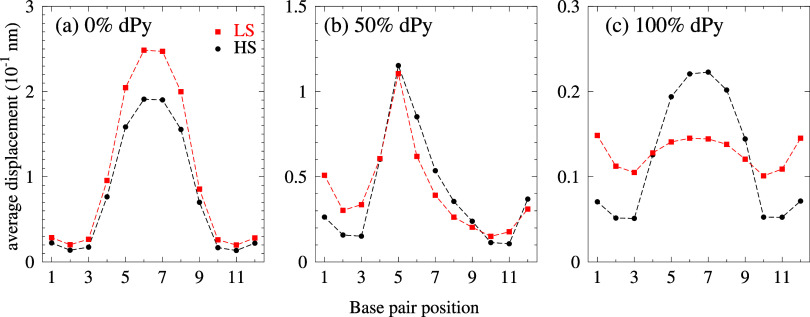
Average displacement profiles for LS (red boxes) and HS (black bullets) parameters for DR. HS profiles were calculated with parameters from^49^. Panel (a) is for a sequence with 0% dPy; panel (b) for 50% dPy; and panel (c) for 100% dPy. Note that the vertical axis scale is different for each panel. Sequences are shown in table S7.

**Table 1:** Average Morse Potentials *D* in meV, Calculated for Low Salt (LS) in the Absence of Cosolute DR Base Pairs^a^

base pair	LS	HS^49^
dArU	22(2)	28(3)
dTrA	41.1(6)	40(2)
dCrG	62(1)	74(1)
dGrC	68(1)	63(1)

a High Salt (HS) Parameters, from^49^, are Shown for Comparison. Standard Deviation is Shown in Compact Uncertainty Notation.

**Table 2:** Average Staking Potentials *k* in eV/nm^2^, Calculated for Low Salt (LS) in the Absence of Cosolute for DR NNs^a^

NN	LS	HS^49^		LS	HS^49^
dArU–dArU	0.9(2)	0.9(5)	dGrC–dArU	2.3(1)	2.4(4)
dArU–dCrG	8.0(5)	2.8(3)	dGrC–dCrG	2.01(9)	2.8(2)
dArU–dGrC	2.7(2)	2.8(4)	dGrC–dGrC	1.3(1)	2.6(2)
dArU–dTrA	4.9(4)	3.1(6)	dGrC–dTrA	1.9(1)	4.3(4)
dCrG–dArU	3.6(2)	2.6(3)	dTrA–dArU	1.3(2)	0.8(4)
dCrG–dCrG	2.7(1)	3.1(3)	dTrA–dCrG	3.1(1)	2.5(3)
dCrG–dGrC	1.41(7)	1.6(1)	dTrA–dGrC	2.1(1)	2.2(2)
dCrG–dTrA	2.6(1)	3.1(3)	dTrA–dTrA	2.5(1)	2.4(2)

a High Salt (HS) Parameters, from^49^, are Shown for Comparison. Standard Deviation is Shown in Compact Uncertainty Notation.

With the new DR-LS results, we have the necessary low salt parameters that will allow us to compare and discuss the results in PEG. The PEG optimization rounds were carried out independently for the DD, DR, and RR data sets. In total, four rounds with 1000 different initial conditions were performed as described in the methods section. Quality parameters for final ⟨Δ*T*⟩ values are between 0.7 and 1.0 °C, which is of the same order of magnitude as found in previous calculations.^49,63,64^

In the following, we will present our results for Morse potentials and stacking elastic constants. Note that we are presently unable to discuss our finding relative to other experimental structural results in PEG since there are none on this subject, to the best of our knowledge. Instead, we will calculate the average displacements as those can be compared to published molecular dynamics simulations, where we then will present our comparative discussion.

In Figure 2, we present the Morse potential depth values *D* for the PEG200 sets, also shown in Table S8. For comparison, the NC values of our previous result for DD^50^ and RR,^51^ and our new DR-LS result are used to visualize the effect of PEG200 for each base pair type. For DD, Figure 2a, we obtained a small reduction of the Morse potentials upon addition of PEG200, suggesting that the hydrogen bond is not sensitive to changes in the number of water molecules present in the minor and major groove. For the DR hybrid, Figure 2b, only the dArU hydrogen bond appears to be unaffected by PEG. In contrast, for dGrC PEG, we observe a small increase. The Morse potentials for RR, Figure 2c, are strongly altered upon addition of PEG. While for rArU we observe an important reduction of *D*, for rCrG we observe a substantial increase on a similar scale as for a LNA-modified C+G.^42^ In comparison to ionic effects, PEG has a much stronger effect on RR hydrogen bonds than Na^+^ at any concentration, see Figure 4a of ref 51.

**Figure 2:**
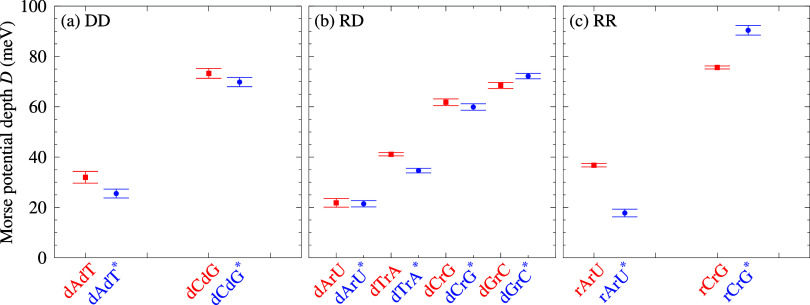
Average Morse potential depths *D* calculated for (a) DD, (b) DR and (c) RR, in PEG200 (blue circles and starred labels) and with NC (red boxes). The NC potentials are from ref 50. for DD; ref 58. for RR; this work for DR (DR-LS). Error bars were obtained from the standard deviation, and were laterally enlarged as to visually highlight the differences.

In Figures 3–5 (also table S9), we show the PEG-related elastic constants *k* calculated for DD, DR and RR, respectively. The elastic constants are ordered to increasing difference to their equivalent NC *k*, the shaded areas in Figures 3–5 highlights those NNs where this difference is larger than 0.5 eV/nm^2^. For DD, Figure 3, only four NN configurations are significantly changed by the presence of PEG, two of which contain only AT base pairs. In particular, for dTdA-dAdT the stacking is found to be severely reduced. Still, this accounts for a large reduction in the Pearson correlation between NC and PEG to 0.41. There are some cases when reduced water activity can lead to a B to A transition in DD, for instance in the presence of trifluoroethanol (TFE)^65^ since the cations are pushed toward the phosphate backbone. However, PEG does form complexes with the cations^25^ and a change in helical structure is not expected. To estimate if there could be any sign of B to A helix transition, we calculated the Pearson correlation of DD in PEG with RR in NC. The rationale for this is that stacking interactions should have some relation to the helix type. In other words, if PEG induces a B to A transition this should somehow be reflected in the change of stacking parameters. An example of this is DR in high salt concentrations where we observed a Pearson correlation of ρ = 0.71 to RR^49^ confirming that DR has a A-type helix.^66^ Since we do not have stacking parameters for A-DNA, we use A-RNA as the closest analogue. The Pearson correlation coefficient calculated in this way was −0.45, that is, a weak anticorrelation of DD in PEG to RR in NC. While this does not preclude a change in helical structure, it appears unlikely that B to A transition occurs in the presence of PEG.

**Figure 3:**
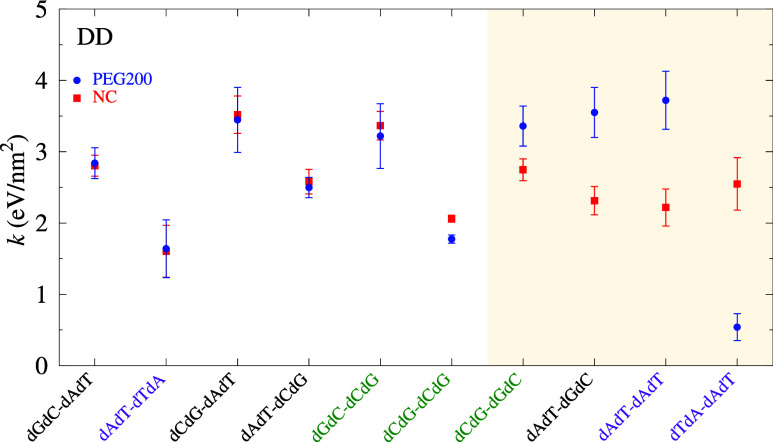
Average stacking interaction *k* calculated for DD with PEG200 (blue bullets) and NC (from^50^, red boxes). Data points are shown in order of increasing difference between PEG200 and NC, and the shaded area mark those where this difference is larger than 0.5 eV/nm^2^. Error bars are the calculated standard deviation of the final minimization round. For visualization, the labels CG-only pair dimers are marked in green, AT-only pair dimers are marked in purple.

For DR, Figure 4, roughly half of the NN configurations show a large change in the presence of PEG. In particular, no NN configurations containing CG only (green labels) show any important change in stacking with PEG. The Pearson correlation between NC and PEG for DR is 0.79, meaning that the stacking interaction largely maintains its A-type helical configuration when PEG is added. From quantum-mechanical calculations of DR by Mignon,^67^ it was concluded that the hydrogen-bonding capacity should increase with electrostatic repulsion between the stacked bases. However, we do not observe important changes in hydrogen bonding, Figure 2, despite some strong changes in stacking shown in Figure 4.

**Figure 4:**
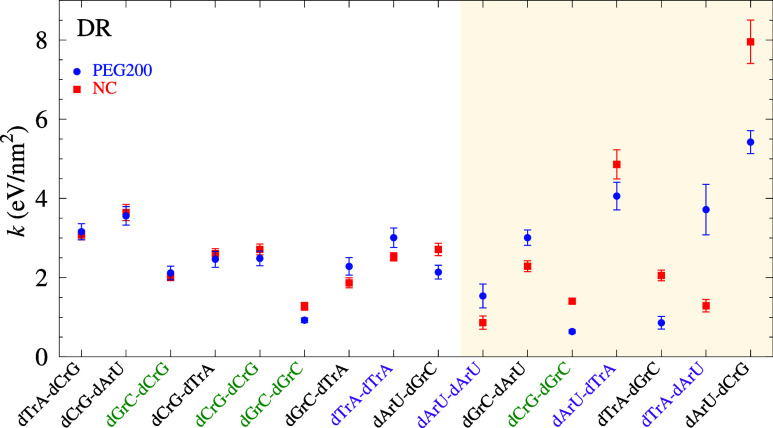
Average stacking interaction *k* calculated for DR with PEG200 (blue bullets) and NC (DR-LS, red boxes). Data points are shown in order of increasing difference between PEG200 and NC, and the shaded area mark those where this difference is larger than 0.5 eV/nm^2^. For visualization, the labels CG-only pair dimers are marked in green, AT/U-only pair dimers are marked in purple.

Most NN configurations for RR, Figure 5, are significantly impacted by the addition of PEG. Interestingly, the three largest changes are for AU-only base pairs, between 3 eV/nm^2^ and 7 eV/nm^2^, which is extremely large. Notice that this contrasts with the strong reduction of AU hydrogen bonds shown in Figure 2c. On the other hand, CG-only stacking for RR (green labels in Figure 2c) is reduced by PEG in opposition to the strong increase of CG hydrogen bonding. RR also has the smallest Pearson correlation of 0.37 between NC and PEG, albeit not as distant as from 0.41 for DD. The strong alterations with PEG in RNA are consistent with the observation of stronger hydration in RR,^9,68,69^ that is, being more hydrated than DD or DR, the reduction of water activity by the addition of PEG is also more likely to alter the intramolecular interactions of RR leading to a greater destabilization of CG-rich duplexes.^23^ However, our results point to a much more intricate picture of the effect of reducing water activity by showing a simultaneous reduction of CG stacking with an increase in the level of CG hydrogen bonding.

**Figure 5:**
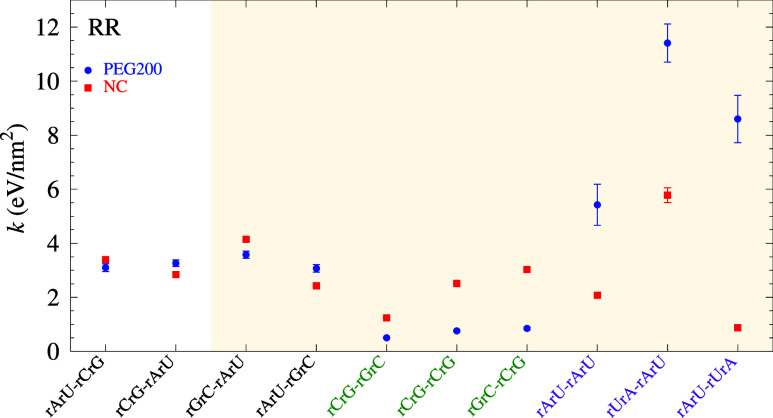
Average stacking interaction *k* calculated for DR with PEG200 (blue bullets) and NC (from^51^, red boxes). Data points are shown in order of increasing difference between PEG200 and NC, and the shaded area mark those where this difference is larger than 0.5 eV/nm^2^. Error bars are the calculated standard deviation of the final minimization round. For visualization, the labels CG-only pair dimers are marked in green, AU-only pair dimers are marked in purple.

The larger effect of PEG200 in RR occurs despite the smaller reduction of melting temperatures, when compared to DD. For DD the average temperature reduction with PEG200 is 14 °C (Table S1), while for RR it is only 8 °C (Table S3). However, we note that in case of RR the much higher hydrogen bonding for CG was compensated by the reduced bonding for AT.

The new parameters in PEG can now be used to calculate average opening profiles ⟨*y*_*i*_⟩ indicating the *i*th base pair in the sequence that is more closely bounded. In Figure 6, we show the calculated average displacement profile for a DD sequence that was studied by molecular dynamics^30^ at two different calculation temperatures 190 and 210 K. At the lower temperatures both curves are nearly identical, but at the higher temperature there is already an onset of dissociation represented by the higher displacement for PEG200. Interestingly, for PEG200, there is a larger displacement at the ends of the sequence, which is similar to what was reported by Mathur and Singh.^30^ However, one need to point out that the findings reported by Mathur and Singh^30^ could be specific to the single DD sequence they studied, as we will see in the next examples.

**Figure 6:**
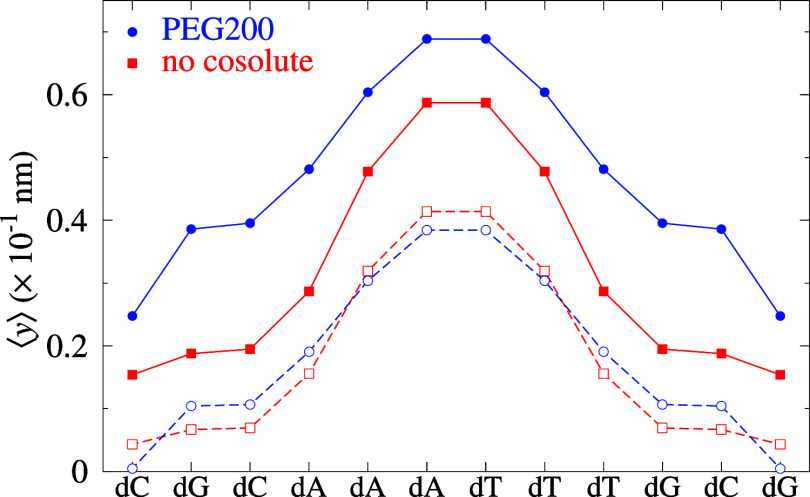
Average displacement profiles the DD sequence CGCAAATTTGCG analyzed by Mathur and Singh.^30^ Blue bullets (circles) are for PEG200 and red filled boxes (squares) for NC at calculation temperature of 210 K (190 K), which is unrelated to the predicted melting temperature.

Now that we have the complete PEG200 parameter sets for DD, DR, and RR, we are in position to compare equivalent sequences. By equivalent, we mean the same CG content and sequence positions. In Figure 7, we show the calculated average displacement profile for four equivalent DD, DR, RD, and RR sequences. The RD sequence is the DR sequence with the ribose and deoxyribose strands switched, that is, the DR duplex is d­(TCCGAATTATCT)/r­(AGGCUUAAUAGA), and the RD is r­(UCCGAAUUAUCU)/d­(AGGCTTAATAGA). The sequences were studied by Banerjee et al.,^45^ which has an rPu content of 33% for RD and 67% for DR. For the hybrid sequences, Figure 7a,b, the addition of PEG has little effect on the opening profiles. In contrast, for RR, Figure 7c, and DD, Figure 7d, PEG200 induces an important destabilization with large base pair openings. Note that for DD, in this case, the effect is larger in the AT region, which is different from what we observed in Figure 6, which suggests that the PEG200 effects may be more sequence-specific than concluded by Mathur and Singh.^30^

**Figure 7:**
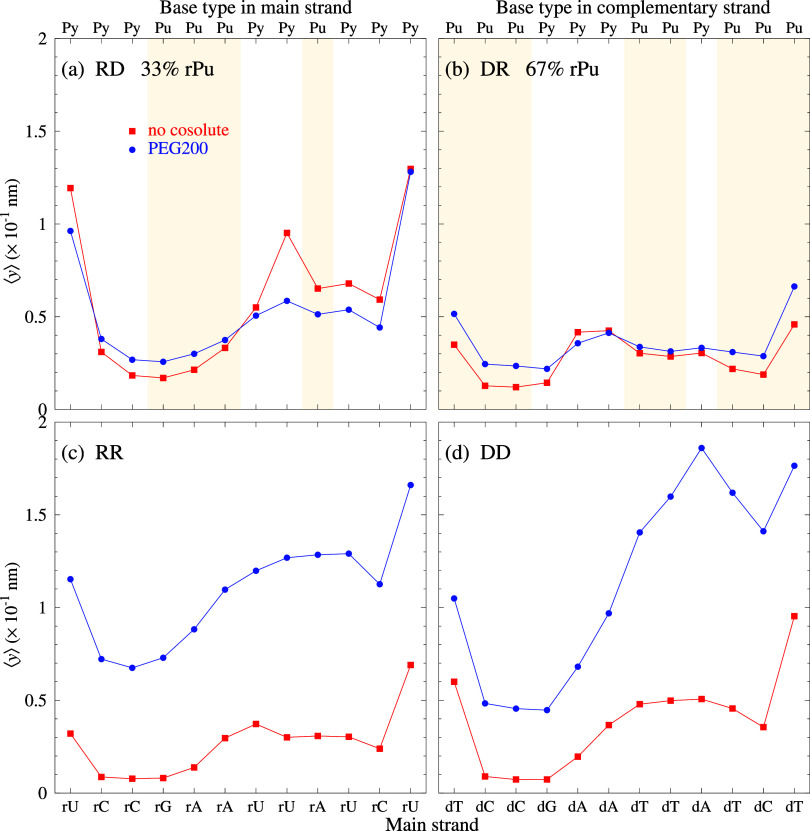
Average displacement profiles for (a) RD sequences from^45^, and their equivalent sequences for (b) DR, (c) RR, and (d) DD. Red boxes are for NC and blue bullets for PEG200. The main strand is displayed at the bottom axis, the complete duplex sequences are shown in table S10. The top axis shows the purine (Pu) and pyrimidine (Py) classification for the main strand in panel (a) and complementary strand in panel (b). The shaded area highlights RNA purine (rPu) regions in panels (a) and (b).

According to Ghosh et al.^22^ and Banerjee et al.,^45^ the measurements in Na^+^ do not represent the cell-like environment which has predominantly K^+^ as monovalent cation. Therefore, they remeasured some of their sequences in a K^+^ buffer and then tested their new NN-model parameters to verify if the predictions were still accurate enough to be valid for this type of environment. The melting temperatures in K^+^ (Tables S4 and S5) are typically 1–2 °C lower than then in Na^+^ buffers. This means that any prediction that is accurate enough for Na^+^ will overshoot those for K^+^ by roughly 2 °C. Expressing this difference in terms of ⟨Δ*T* ⟩, eq 6, between the melting temperatures in K^+^ and Na^+^ buffer, we obtain 2.25 °C for RR and 1.95 °C for DR. For the new PB parameters, we obtain very similar quality parameters ⟨Δ*T* ⟩= 2.18 °C for RR and 2.19 °C and DR, shown in Table S11.

## 4 Conclusions

Here, we calculated the effects of hydrogen bonds and stacking interactions in the presence of PEG200 in DD, DR, and RR duplexes. To achieve this, we used a mesoscopic model to extract this type of information from 126 experimental melting temperatures, following a well established parameter optimization procedure.^36,39,42–44,49,51,63^ We found that hydrogen bonding is strongly modified for RR, in particular, CG in PEG200 becomes almost as strong as a LNA modification.^42^ In contrast, for DD and DR, there is little change in the Morse potentials, which are representative of hydrogen bonding. This means that the addition of PEG is really very neutral for hydrogen bonding in DD and DR and therefore should not influence the recognition potential of DD and DR.

The Pearson correlation of the stacking interaction with and without PEG was calculated to evaluate the possibility of changes in the type of the helix. In particular, the changes of stacking interactions for DD do not exhibit any tendency of B-DNA to A-DNA transition since they do not correlate at all to A-RNA. The new parameters allowed us to calculate base pair displacement profiles comparing the effect of PEG in equivalent DD, DR, and RR duplexes. These displacement profiles show that the general tendency of PEG is to destabilize the double helix, as shown by the displacement profiles. The destabilization occurs despite the strong CG hydrogen bonding found for RR, which appears to be compensated by very low CG-CG stacking.

## Supplementary Material



## Notes

Tables S1–S3 show the sequences and temperatures in 40 wt % PEG200 obtained from published sources. Tables S4 and S5 show the sequences and temperatures measured in intracellular cation condition. Table S6 shows the DR low salt melting temperatures obtained from published sources. Table S7 shows the sequences used for Figure 1. Tables S8 and S9 compares the Morse and stacking potentials, respectively, for DD, DR, and RR. Table S10 shows the sequences used for the calculation of the average displacement profiles. Table S11 shows the quality parameters of the melting temperatures predictions in intracellular cation condition for the Peyrard-Bishop and Nearest-Neighbors Models (PDF)
