# Supplementary material for: Effects of Polyethylene Glycol on the Hydrogen Bonds and Stacking Interactions of DNA/DNA, DNA/RNA, and RNA/RNA Calculated by a Mesoscopic Model
Source: ACS Omega. 2026 May 12;11(20):29787–95. doi: 10.1021/acsomega.6c00104 (PMC13216965; doi:10.1021/acsomega.6c00104)
Supplement: Supplementary file 1 [file ao6c00104_si_001.pdf]

Supplementary Information: Effects of polyethylene glycol on the hydrogen bonds and stacking interactions of DNA/DNA, DNA/RNA and RNA/RNA calculated by a mesoscopic model

Luciano Gabriel Silva[a], Gerald Weber[a,*]

[a]*Departamento de Física, Universidade Federal de Minas Gerais, 31270-901, Belo Horizonte-MG, Brazil*

The software is available for multiple Linux distribution. All data files and software can be obtained from one of the following sources:

- `https://bioinf.fisica.ufmg.br/software`
- `https://sites.google.com/site/geraldweberufmg/tfreg`
- `https://software.opensuse.org//download.html?project=home%3Adrgweber&package=TfReg`
- `http://download.opensuse.org/repositories/home:/drgweber/`

List of Tables

S1 DD melting temperatures. 2
S2 DR melting temperatures. 3
S3 RR melting temperatures. 4
S4 Melting temperature comparison to K^+ buffer for DR. 5
S5 Melting temperature comparison to K^+ buffer for RR. 5
S6 DR low salt melting temperatures. 6
S7 DR sequences from Ref. 1 used for the calculation of average displacement profiles. 9
S8 Comparison of Morse potentials. 10
S9 Comparison of elastic constants. 10
S10 Sequences used for the calculation of average displacement profiles. 11
S11 Quality parameters of experimental, NN and PB predictions in K^+ buffer. 11

*Corresponding author
Email address: `gweberbh@gmail.com` (Gerald Weber)

Table S1: DD sequences used in this work. T are the measured temperature, T' the calculated melting temperatures using the PB model with the new parameters and T'(NC) the no cosolute calculated melting temperatures using the NN model using the SantaLucia et al. [2] parameters. The column labeled stability reduction is the difference between T'(NC) and T.

				PEG200		NC	Stability reduction
Sequence (5′ → 3′)	Ref.	C_t (μM)	[Na$^+$] (mM)	T (°C)	T' (°C)	T' (°C)	$\Delta T'$ (°C)
GGACGTCC	3, 4	100	122	35.40	35.73	52.82	–17.42
GACCGGTC	3, 4	100	122	35.60	35.73	52.82	–17.22
CGTCGACG	3, 4	100	122	39.00	39.37	53.97	–14.97
CGACGTCG	3, 4	100	122	39.30	39.37	53.97	–14.67
CAAGCTTG	3, 4	100	122	28.90	29.52	38.13	–9.23
CTTGCAAG	3, 4	100	122	29.60	29.52	38.13	–8.53
CGGTACCG	3, 4	100	122	32.30	33.76	52.10	–19.80
CCGTACGG	3, 4	100	122	34.70	33.76	52.10	–17.40
GATCCGGATC	3, 4	100	122	37.30	38.32	53.79	–16.49
GGATCGATCC	3, 4	100	122	38.20	38.32	53.79	–15.59
ATGAGCTCAT	3, 4	100	122	34.30	34.68	45.20	–10.90
ATCAGCTGAT	3, 4	100	122	34.00	34.68	45.20	–11.20
CATAGGCCTATG	3, 4	100	122	39.80	40.28	52.53	–12.73
CTATGGCCATAG	3, 4	100	122	40.50	40.28	52.53	–12.03
AGTCATGACT	3, 4	100	122	32.30	32.62	45.16	–12.86
GCGAATTCGC	3, 4	100	122	43.10	42.15	56.90	–13.80
ATCGCTAGCGAT	3, 4	100	122	43.10	44.03	58.32	–15.22
GACGACGTCGTC	3, 4	100	122	48.20	50.71	66.15	–17.95
GCAAGCCGGCTTGC	3, 4	100	122	58.50	57.25	75.23	–16.73
CGATCGGCCGATCG	3, 4	100	122	56.90	57.13	74.64	–17.74
CATATGGCCATATG	3, 4	100	122	40.70	41.34	54.90	–14.20
CAAGATCGATCTTG	3, 4	100	122	44.60	44.72	56.58	–11.98
CGCGTACGCGTACGCG	3, 4	100	122	57.90	57.14	78.37	–20.47
CGCAAGCCGGCTTGCG	3, 4	100	122	64.20	62.32	81.02	–16.82
GGCAGTTC	4	200	122	32.45	32.60	45.57	–13.12
GGTTCAGC	4	200	122	31.98	32.61	45.57	–13.59
CGCTGTAG	4	200	122	33.64	32.95	42.82	–9.18
CGTGCTAG	4	200	122	32.94	32.95	42.82	–9.88
AGTAACGCCAT	4	200	122	40.00	39.79	53.82	–13.83
AATGCCGTAGT	4	200	122	39.40	39.79	53.82	–14.43
CCATCGCTACC	4	200	122	44.81	44.66	59.79	–14.98
CGATGGCCTAC	4	200	122	45.64	44.66	59.79	–14.15
CGCTTGTTAC	4	200	122	37.39	35.28	49.37	–11.97
CCGTAACGTTGG	4	200	122	42.30	45.94	61.98	–19.68
ACTGACTGACTG	4	200	122	43.80	42.82	56.45	–12.64
ACTGACTGACTGACTG	4	200	122	52.10	50.87	65.81	–13.71
							average reduction:–14.36

Table S2: DR sequences used in this work. T are the measured temperature, T' the calculated melting temperatures using the PB model with the new parameters and T'(NC) the no cosolute calculated melting temperatures using the NN model using th Banerjee et al. [5] parameters. The column labeled stability reduction is the difference between T'(NC) and T.

				PEG200		NC	Stability reduction
Sequence ($5' \to 3'$)	Ref.	C_t (μM)	[Na$^+$] (mM)	T (°C)	T' (°C)	T' (°C)	$\Delta T'$ (°C)
r(GCCGUGAG)	6	100	122	38.28	37.54	48.91	–10.64
r(GAGCCGUG)	6	100	122	37.86	37.54	48.91	–11.05
r(AAUCUGGCCA)	6	100	122	40.95	39.27	47.68	–6.73
r(AUGGCUCCAA)	6	100	122	38.71	39.27	47.68	–8.97
r(GGCAGGAAUCCG)	6	100	122	52.28	51.32	61.96	–9.68
r(GGAAUCAGGCCG)	6	100	122	52.19	51.33	61.96	–9.76
r(CGGAUUCCUGCC)	6	100	122	49.67	49.97	56.88	–7.21
r(CGGCCUUGAUCC)	6	100	122	48.98	49.96	56.88	–7.91
r(AAUGGAUUACAA)	6	100	122	29.97	30.11	42.06	–12.09
r(AUUGGAUACAAA)	6	100	122	31.09	30.11	42.06	–10.96
r(UAUCUUCCGAAU)	6	100	122	29.68	31.55	38.94	–9.26
r(UAUCCUUCGAAU)	6	100	122	30.04	31.55	38.94	–8.90
r(AUUCGGAAGAUA)	6	100	122	35.86	34.25	44.38	–8.52
r(AUUAGGAUCGAA)	6	100	122	34.77	34.25	44.38	–9.61
r(GUUAGCGUUACGC)	6	100	122	43.59	44.27	50.79	–7.21
r(GCGUUUACGUAGC)	6	100	122	44.66	44.27	50.79	–6.14
r(GCGAUCGGA)	6	100	122	38.87	39.08	52.50	–13.62
r(GCCAGUAGG)	6	100	122	39.41	40.71	50.28	–10.87
r(GUUCAAUACG)	6	100	122	25.35	23.42	36.15	–10.80
r(AGGAUGACCG)	6	100	122	42.51	42.36	55.63	–13.12
r(GGGGAACAAGG)	6	100	122	48.81	50.90	60.65	–11.83
r(UUCACCUGGUC)	6	100	122	43.07	41.99	52.78	–9.71
r(CCUGGAAUCCAA)	6	100	122	44.01	43.85	54.98	–10.96
r(GGCUCAAUUGAC)	6	100	122	41.84	43.31	51.79	–9.95
r(GAAGAGAGAAGC)	6	100	122	49.09	49.08	57.54	–8.45
r(CUGAAUACCAAUG)	6	100	122	38.35	38.36	49.56	–11.20
r(UCACGUAGUCGUAU)	6	100	122	43.65	43.80	55.63	–11.98
r(CACUGAAUACCAAUGC)	6	100	122	45.35	45.84	59.26	–13.91
r(CGCUUGUUAC)	6	100	122	31.45	30.62	36.56	–5.11
r(GUAACAAGCG)	6	100	122	32.75	34.37	45.70	–12.95
r(UCCGAAUUAUCU)	6	100	122	32.98	31.55	38.94	–5.96
r(AGAUAAUUCGGA)	6	100	122	31.48	34.25	44.38	–12.90
r(UCGUUCUUGUCU)	6	100	122	34.57	35.79	41.89	–7.33
r(AGACAAGAACGA)	6	100	122	39.89	40.74	53.43	–13.54
r(GAGAGAAAGG)	6	100	122	40.88	40.08	50.99	–10.10
r(GAAGAGAGAAGG)	6	100	122	46.24	47.44	57.94	–11.70
r(GGAGGAAAGGAG)	6	100	122	56.03	52.99	61.96	–5.93
r(CAAGAGAGAACC)	6	100	122	45.61	45.21	56.02	–10.41
r(CAAGAGAGAAGC)	6	100	122	46.09	46.44	56.26	–10.17
r(CUCCUUUCCUCC)	6	100	122	41.30	41.62	43.45	–2.15
r(GGUUCUCUCUUG)	6	100	122	37.84	39.55	43.78	–5.94
r(GCUUCUCUCUUG)	6	100	122	37.84	38.04	39.08	–1.24
							average reduction:–9.44

Table S3: RR sequences used in this work. T are the measured temperature, T' the calculated melting temperatures using the PB model with the new parameters and T'(NC) the no cosolute calculated melting temperatures using the NN model using the Ferreira et al. [7] parameters. The column labeled stability reduction is the difference between T'(NC) and T.

				PEG200		NC	Stability reduction
Sequence (5′ → 3′)	Ref.	C_t (μM)	[Na^+] (mM)	T (°C)	T' (°C)	T' (°C)	$\Delta T'$ (°C)
CAUGCC	8	200	122	34.26	33.08	38.72	–4.46
CUAGGC	8	200	122	36.98	34.51	43.45	–6.47
CAGCGG	8	200	122	38.41	39.01	48.26	–9.85
CUACGC	8	200	122	30.22	31.61	40.14	–9.92
GAACUCC	8	200	122	36.12	35.81	46.07	–9.95
GGCUGUCC	8	200	122	55.95	56.79	66.05	–10.11
GGCUGUUC	8	200	122	47.43	47.48	56.40	–8.97
GGUUCUGC	8	200	122	46.55	47.48	56.40	–9.85
AGCUGUCU	8	200	122	44.42	44.70	54.34	–9.92
AGUCUGCU	8	200	122	45.25	44.70	54.34	–9.09
GGCAGUUC	8	200	122	48.09	47.48	56.40	–8.31
GGUUCAGC	8	200	122	47.25	47.48	56.40	–9.15
CGCUGUCG	8	200	122	52.35	52.46	59.38	–7.04
CGUCUGCG	8	200	122	52.54	52.46	59.38	–6.85
UGCUGUCA	8	200	122	45.16	45.30	53.14	–7.98
UGUCUGCA	8	200	122	45.27	45.30	53.14	–7.87
UAUGAGGA	8	200	122	36.55	36.41	45.18	–8.63
UAGAUGGA	8	200	122	36.98	36.41	45.18	–8.20
GUGCCGAG	8	200	122	56.02	54.64	62.77	–6.75
GCCGAGUG	8	200	122	56.80	54.64	62.77	–5.97
CGCUGUAG	8	200	122	46.28	46.79	54.82	–8.54
CGUGCUAG	8	200	122	47.57	46.79	54.82	–7.24
AUUGGAUACAAA	8	200	122	43.88	42.81	49.74	–5.86
AUACAUUGGAAA	8	200	122	42.87	42.81	49.74	–6.87
GGCUCAAUUGAC	8	200	122	57.51	57.86	63.64	–6.13
UACAUGUA	8	100	122	27.06	26.71	36.37	–9.31
GGUAUACC	8	100	122	39.28	40.02	51.80	–12.52
GGUUAACC	8	100	122	37.10	37.19	49.42	–12.32
CGAAUUCG	8	100	122	31.90	32.91	39.16	–7.26
GCUUAAGC	8	100	122	38.88	38.45	47.99	–9.11
CGUAUACG	8	100	122	36.26	35.51	44.11	–7.85
CGCAUGCG	8	100	122	53.15	52.53	57.18	–4.04
CGGUACCG	8	100	122	51.71	52.14	62.72	–11.01
CAAGCUUG	8	100	122	35.52	37.41	43.98	–8.46
CUUGCAAG	8	100	122	38.30	37.41	43.98	–5.68
GAACGUUC	8	100	122	37.10	35.44	45.33	–8.23
GUUCGAAC	8	100	122	35.23	35.44	45.33	–10.10
GAUCCGGAUC	8	100	122	58.12	57.29	62.28	–4.16
GGAUCGAUCC	8	100	122	55.81	57.28	62.28	–6.47
AUGAGCUCAU	8	100	122	48.66	48.79	54.28	–5.62
AUCAGCUGAU	8	100	122	46.69	48.79	54.28	–7.59
UUACGCGUAA	8	100	122	43.11	43.63	54.06	–10.95
AUCGCUAGCGAU	8	100	122	60.18	59.78	66.82	–6.64
CAUAGGCCUAUG	8	100	122	59.15	59.31	66.01	–6.86
CUAUGGCCAUAG	8	100	122	59.27	59.31	66.01	–6.73
							average reduction:–8.02

Table S4: Comparison between measured and predicted melting temperatures in K^+ buffer for the DR sequences from [6]. EXP are the measured temperature in K^+ from [6]; NN are the predicted temperature in Na^+ buffer for NN model parameters from [6]; PB are the predicted temperature in Na^+ buffer for the new PB model parameters; RNA strands shown are in 5′ to 3′ direction and all temperatures are in °C.

	temperatures		
Sequence	EXP	NN	PB
r(CGGAUUCCUGCC)	47.60	49.67	49.97
r(AUUCGGAAGAUA)	33.80	34.86	34.25
r(GGCUCAAUUGAC)	39.60	41.29	43.31
r(UCACGUAGUCGUAU)	42.10	43.09	43.80
r(CGCUUGUUAC)	29.50	30.22	30.62
r(GUAACAAGCG)	31.30	33.94	34.37
r(UCGUUCUUGUCU)	32.20	36.33	35.79
r(CACUUGUUAC)	26.20	25.52	24.82

Table S5: Comparison between measured and predicted melting temperatures in K^+ buffer for the RR sequences from [8]. EXP are the measured temperature in K^+ from [8]; NN are the predicted temperature in Na^+ buffer for NN model parameters from [8]; PB are the predicted temperature in Na^+ buffer for the new PB model parameters; Strands shown are in 5′ to 3′ direction and all temperatures are in °C.

	temperatures		
Sequence	EXP	NN	PB
r(CAGCGG)	36.00	37.07	39.01
r(GAACUCC)	35.10	34.57	35.81
r(CGCUGUCG)	48.70	51.00	52.46
r(UGCUGUCA)	41.60	44.55	45.30
r(GUGCCGAG)	53.00	53.37	54.64
r(GCCGAGUG)	53.60	53.37	54.64
r(GGUAUACC)	37.90	40.90	40.02
r(GGUUAACC)	35.40	37.97	37.19
r(GCUUAAGC)	35.40	39.22	38.45
r(CGCAUGCG)	50.30	52.14	52.53
r(CGGUACCG)	52.00	50.96	52.14
r(GAUCCGGAUC)	57.00	57.51	57.29
r(AUGAGCUCAU)	46.80	48.76	48.79
r(AUCAGCUGAU)	44.70	48.76	48.79
r(UUACGCGUAA)	41.70	42.72	43.63
r(AUCGCUAGCGAU)	57.10	60.18	59.78

Table S6: DR low salt melting temperatures. T are the measured temperature and T' the calculated melting temperatures using the PB model with the new DR-LS parameters.

Sequence ($5' \rightarrow 3'$)	Ref.	C_t (μM)	[Na^+] (mM)	T (°C)	T' (°C)
r(UCCCUCCUCUCC)	9	8	100	43.40	44.95
r(CCUUCCCUU)	9	8	100	20.50	21.88
r(UUCCCUUCC)	9	8	100	14.90	21.15
r(GCUCUCUGGC)	9	8	100	40.80	42.93
r(CUCGUACCUUCCGGUCC)	9	8	100	56.00	58.10
r(CUCGUACCUUUCCGGUCC)	9	8	100	56.80	55.86
r(UAGUUAUCUCUAUCU)	9	8	100	34.90	35.65
r(GCACAGCC)	9	8	100	35.60	38.02
r(GAGCUCCCAGGC)	9	8	100	56.70	57.27
r(GCCGAGGUCCAUGUCGUACGC)	9	8	100	68.10	66.05
r(UGUACGUCACAACUA)	9	8	100	49.20	49.60
r(UAUACAAGUUAUCUA)	9	8	100	35.90	34.51
r(CGACUAUGCAAAAAC)	9	8	100	47.30	48.59
r(CGCAAAAAAAAAACGC)	9	8	100	50.20	51.35
d(TCCCTCCTCTCC)	9	8	100	61.40	61.70
d(CCTTCCCTT)	9	8	100	44.80	42.17
d(TTCCCTTCC)	9	8	100	44.20	42.89
d(GCTCTCTGGC)	9	8	100	50.90	50.22
d(CTCGTACCTTCCGGTCC)	9	8	100	64.80	65.40
d(CTCGTACCTTTCCGGTCC)	9	8	100	65.20	64.72
d(TAGTTATCTCTATCT)	9	8	100	45.40	43.59
d(GCACAGCC)	9	8	100	37.20	41.20
d(GAGCTCCCAGGC)	9	8	100	60.30	57.19
d(GCCGAGGTCCATGTCGTACGC)	9	8	100	68.20	64.71
d(TGTACGTCACAACTA)	9	8	100	50.60	51.19
d(TATACAAGTTATCTA)	9	8	100	35.20	35.84
d(CGACTATGCAAAAAC)	9	8	100	39.00	41.21
d(CGCAAAAAAAAAACGC)	9	8	100	28.70	29.25
r(GAAGAGAAGC)	10	8	100	46.90	43.24
d(GAAGAGAAGC)	10	8	100	23.70	23.38
d(CGACTATGCAAGTAC)	11	8	100	45.10	49.22
d(GGACCGGAAGGTACGAG)	11	8	100	57.00	58.10

continued on next page

Tab. S6 continued

Sequence (5′ → 3′)	Ref.	C_t (μM)	[Na^+] (mM)	T (°C)	T' (°C)
d(CTCGTACCATTCCGGTCC)	11	8	100	63.70	64.45
r(GCCAGUUAA)	12	8	100	30.60	29.17
r(AUUGGAUACAAA)	12	8	100	35.50	35.01
r(GGUCGC)	5	8	100	27.20	26.64
r(CGGACC)	5	8	100	26.10	24.29
r(GCCGUGAG)	5	8	100	41.20	40.22
r(GAGCCGUG)	5	8	100	41.50	40.22
r(GUCAGACU)	5	8	100	29.70	29.78
r(GACAGUCU)	5	8	100	30.10	29.78
r(GAACUGCC)	5	8	100	33.50	31.43
r(GGCAGUUC)	5	8	100	33.80	32.05
r(GCGAUCGGA)	5	8	100	43.50	41.90
r(GCCAGUAGG)	5	8	100	42.60	42.30
r(GUUCAAUACG)	5	8	100	27.50	28.50
r(AGGAUGACCG)	5	8	100	45.90	45.17
r(CGCUUGUUAC)	5	8	100	33.10	29.48
r(GUAACAAGCG)	5	8	100	39.20	38.85
r(CACUUGUUAC)	5	8	100	28.10	25.19
r(AAUCUGGCCA)	5	8	100	42.80	40.07
r(AUGGCUCCAA)	5	8	100	40.10	40.07
r(GGGGAACAAGG)	5	8	100	54.30	53.54
r(UUCACCUGGUC)	5	8	100	45.30	41.32
r(GGCAGGAAUCCG)	5	8	100	56.80	54.84
r(GGAAUCAGGCCG)	5	8	100	56.30	54.84
r(UAUCUUCCGAAU)	5	8	100	30.20	32.76
r(UAUCCUUCGAAU)	5	8	100	29.60	32.77
r(AAUGGAUUACAA)	5	8	100	36.30	35.01
r(AUUGGAUACAAA)	5	8	100	36.20	35.01
r(CCUGGAAUCCAA)	5	8	100	48.20	46.87
r(GGCUCAAUUGAC)	5	8	100	45.20	43.82
r(CGGCCUUGAUCC)	5	8	100	51.90	49.25
r(CGGAUUCCUGCC)	5	8	100	50.30	49.26
r(UCCGAAUUAUCU)	5	8	100	35.80	32.77
r(AGAUAAUUCGGA)	5	8	100	35.50	38.19
r(GCUUCUCUCUUC)	5	8	100	31.50	31.99
r(GAAGAGAGAAGC)	5	8	100	54.00	51.58

continued on next page

Tab. S6 continued

Sequence (5′ → 3′)	Ref.	C_t (μM)	[Na^+] (mM)	T (°C)	T' (°C)
r(UCGUUCUUGUCU)	5	8	100	36.40	37.01
r(AGACAAGAACGA)	5	8	100	47.60	47.28
r(GUUAGCGUUACGC)	5	8	100	45.00	46.02
r(GCGUUUACGUAGC)	5	8	100	47.80	46.01
r(UCACGUAGUCGUAU)	5	8	100	49.80	50.62

Table S7: DR sequences from Ref. 1 used for the calculation of average displacement profiles.

dPy (%)	DR
0	d(GGGAAAAAAGGG)<br>r(CCCUUUUUUCCC)
50	d(CGCAAATTTGCG)<br>r(GCGUUUAAACGC)
100	d(CCCTTTTTTCCC)<br>r(GGGAAAAAAGGG)

Table S8: Comparison of Morse potentials D in meV. Base pair configurations are grouped by equivalence: dA⟷rA, dT⟷rU, dC⟷rC and dG⟷rG.

DD		DR		RR	
Base pair	D	Base pair	D	Base pair	D
dAdT	26(2)	dArU	21(1)	rArU	18(2)
		dTrA	34.6(9)		
dCdG	70(2)	dCrG	60(1)	rCrG	90(2)
		dGrC	72(1)		

Table S9: Comparison of elastic constants k in eV/nm^2. NN configurations are grouped by equivalence in the same way as in table S8.

DD		DR		RR	
NN	k	NN	k	NN	k
dAdT-dAdT	3.7(4)	dArU-dArU	1.5(3)	rArU-rArU	5.4(8)
		dTrA-dTrA	3.0(2)		
dAdT-dTdA	1.6(4)	dArU-dTrA	4.1(3)	rArU-rUrA	8.6(9)
dTdA-dAdT	0.5(2)	dTrA-dArU	3.7(6)	rUrA-rArU	11.4(7)
dAdT-dCdG	2.5(1)	dArU-dCrG	5.4(3)	rArU-rCrG	3.1(1)
		dGrC-dTrA	2.3(2)		
dAdT-dGdC	3.5(4)	dArU-dGrC	2.1(2)	rArU-rGrC	3.1(1)
		dCrG-dTrA	2.5(2)		
dCdG-dAdT	3.4(5)	dCrG-dArU	3.6(2)	rGrC-rArU	3.6(1)
		dTrA-dGrC	0.9(2)		
dGdC-dAdT	2.8(2)	dGrC-dArU	3.0(2)	rCrG-rArU	3.3(1)
		dTrA-dCrG	3.2(2)		
dCdG-dCdG	1.78(6)	dCrG-dCrG	2.5(2)	rCrG-rCrG	0.76(3)
		dGrC-dGrC	0.93(6)		
dCdG-dGdC	3.4(3)	dCrG-dGrC	0.64(4)	rCrG-rGrC	0.50(2)
dGdC-dCdG	3.2(5)	dGrC-dCrG	2.1(2)	rGrC-rCrG	0.85(6)

Table S10: Sequences from Banerjee et al. [6] used for the calculation of average displacement profiles.

type	sequence (a)	sequence (b)
RD	5′−r(UCCGAAUUAUCU)−3′<br>3′−d(AGGCTTAATAGA)−5′	5′−r(AGAUAAUUCGGA)−3′<br>3′−d(TCTATTAAGCCT)−5′
DD	5′−d(TCCGAATTATCT)−3′<br>3′−d(AGGCTTAATAGA)−5′	5′−d(AGATAATTCGGA)−3′<br>3′−d(TCTATTAAGCCT)−5′
DR	5′−d(TCCGAATTATCT)−3′<br>3′−r(AGGCUUAAUAGA)−5′	5′−d(AGATAATTCGGA)−3′<br>3′−r(UCUAUUAAGCCU)−5′
RR	5′−r(UCCGAAUUAUCU)−3′<br>3′−r(AGGCUUAAUAGA)−5′	5′−r(AGAUAAUUCGGA)−3′<br>3′−r(UCUAUUAAGCCU)−5′

Table S11: Quality parameters of the melting temperatures in K^+ buffer. EXP stands for the comparison between experimental temperatures in Na^+ and K^+ buffer; NN (PB) for the comparison between experimental temperature in K^+ buffer and prediction using NN (PB) model parameters for Na^+ buffer.

Type	Model	$\langle \Delta T \rangle$ (°C)	$\langle \chi^2 \rangle$ (°C^2)
DR	EXP	1.95	3.92
DR	NN	1.75	4.28
DR	PB	2.17	5.99
RR	EXP	2.25	6.09
RR	NN	1.90	5.08
RR	PB	2.13	5.93

References

[1] G. Suresh, U. D. Priyakumar, DNA–RNA hybrid duplexes with decreasing pyrimidine content in the DNA strand provide structural snapshots for the A-to B-form conformational transition of nucleic acids, Phys. Chem. Chem. Phys. 16 (2014) 18148–18155. doi: doi:10.1039/c4cp02478h.

[2] J. SantaLucia, Jr., H. T. Allawi, P. A. Seneviratne, Improved nearest-neighbour parameters for predicting DNA duplex stability, Biochem. 35 (1996) 3555–3562. doi: doi:10.1021/bi951907q.

[3] S. Ghosh, S. Takahashi, T. Endoh, H. Tateishi-Karimata, S. Hazra, N. Sugimoto, Validation of the nearest-neighbor model for Watson–Crick self-complementary DNA duplexes in molecular crowding condition, Nucleic Acids Res. 47 (2019) 3284–3294. URL: http://dx.doi.org/10.1093/nar/gkz071. doi: doi:10.1093/nar/gkz071.

[4] S. Ghosh, S. Takahashi, T. Ohyama, T. Endoh, H. Tateishi-Karimata, N. Sugimoto, Nearest-neighbor parameters for predicting DNA duplex stability in diverse molecular crowding conditions, Proc. Natl. Acad. Sci. USA 117 (2020) 14194–14201. URL: http://dx.doi.org/10.1073/pnas.1920886117. doi: doi:10.1073/pnas.1920886117.

[5] D. Banerjee, H. Tateishi-Karimata, T. Ohyama, S. Ghosh, T. Endoh, S. Takahashi, N. Sugimoto, Improved nearest-neighbor parameters for the stability of RNA/DNA hybrids under a physiological condition, Nucleic Acids Res. 48 (2020) 12042–12054. doi: doi:10.1093/nar/gkaa572.

[6] D. Banerjee, H. Tateishi-Karimata, M. Toplishek, T. Ohyama, S. Ghosh, S. Takahashi, M. Trajkovski, J. Plavec, N. Sugimoto, In-cell stability prediction of RNA/DNA hybrid duplexes for designing oligonucleotides aimed at therapeutics, J. Am. Chem. Soc. (2023). URL: https://doi.org/10.1021/jacs.3c06706. doi: doi:10.1021/jacs.3c06706.

[7] I. Ferreira, T. D. Amarante, G. Weber, Salt dependent mesoscopic model for RNA with multiple strand concentrations, Biophys. Chem. 271 (2021) 106551. doi: doi:10.1016/j.bpc.2021.106551.

[8] S. Ghosh, S. Takahashi, D. Banerjee, T. Ohyama, T. Endoh, H. Tateishi-Karimata, N. Sugimoto, Nearest-neighbor parameters for the prediction of RNA duplex stability in diverse *in vitro* and cellular-like crowding conditions, Nucleic Acids Res. (2023). URL: https://doi.org/10.1093%2Fnar%2Fgkad020. doi: doi:10.1093/nar/gkad020.

[9] E. A. Lesnik, S. M. Freier, Relative thermodynamic stability of DNA, RNA, and DNA: RNA hybrid duplexes: relationship with base composition and structure, Biochem. 34 (1995) 10807–10815. doi: doi:10.1021/bi00034a013.

[10] J. I. Gyi, G. L. Conn, A. N. Lane, T. Brown, Comparison of the thermodynamic stabilities and solution conformations of DNA·RNA hybrids containing purine-rich and pyrimidine-rich strands with DNA and RNA duplexes, Biochem. 35 (1996) 12538–12548. doi: doi:10.1021/bi960948z.

[11] A. M. Kawasaki, M. D. Casper, S. M. Freier, E. A. Lesnik, M. C. Zounes, L. L. Cummins, C. Gonzalez, P. D. Cook, Uniformly modified

2′-deoxy-2′-fluoro-phosphorothioate oligonucleotides as nuclease-resistant antisense compounds with high affinity and specificity for RNA targets, J. Med. Chem. 36 (1993) 831–841. doi: doi:10.1021/jm00059a007.
[12] S. Nakano, M. Fujimoto, H. Hara, N. Sugimoto, Nucleic acid duplex stability: influence of base composition on cation effects, Nucleic Acids Res. 27 (1999) 2957. doi: doi:10.1093/nar/27.14.2957.
